# Risk and Protective Factors for Cognitive Reserve in Oldest-Old Japanese Americans

**DOI:** 10.1093/geroni/igaa057.2822

**Published:** 2020-12-16

**Authors:** Bradley Willcox, Randy Chen, Tim Donlon, D Craig Willcox, Richard Allsopp, Kamal Masaki

**Affiliations:** 1 Kuakini Health Systems, Honolulu, Hawaii, United States; 2 Kuakini Medical Center, Honolulu, Hawaii, United States; 3 University of Hawaii, Honolulu, Hawaii, United States; 4 Okinawa International University, Urasoe, Okinawa, Japan

## Abstract

3,734 Japanese-American male oldest-old (aged 85+ years), from the Kuakini Honolulu Asia Aging Study, were assessed for prevalent cognitive impairment (CI). CI was defined as scoring <74 on the 100-point Cognitive Abilities Screening Instrument (CASI; 80% sensitivity, 90% specificity for dementia). CASI tests from 1991 (Exam 4) to 2012 (Exam 12) identified 1496 cases of CI (i.e. low cognitive reserve) and 1222 non-CI controls (mean diagnosis age: 85.7 ± 5.3; range 71-100 years). Baseline risk factors were compared between groups, adjusted for age at onset of CI or last CASI. Step-wise conditional logistic regression was used to assess risk for CI. Among other factors, education (0.88; 0.85-0.9, p<0.0001), hand-grip strength (0.98; 0.97-0.99, p=0.03), and height (0.97; 0.96-0.99, p=0.002) had protective effects; whereas APOE4 carriage (1.31; 1.04-1.63, p=0.02) and depression (1.4; 1.06-1.96, p=0.02) were risk factors for CI. The implications of these data for cognitive and physical health will be discussed.

